# Supplementary material for: Association of Metabolically Healthy Obesity and Future Depression: Using National Health Insurance System Data in Korea from 2009–2017
Source: Int J Environ Res Public Health. 2020 Dec 23;18(1):63. doi: 10.3390/ijerph18010063 (PMC7795335; doi:10.3390/ijerph18010063)
Supplement: Supplementary file 1 [file ijerph-18-00063-s001.pdf]

Supplementary Figure S1. Study population

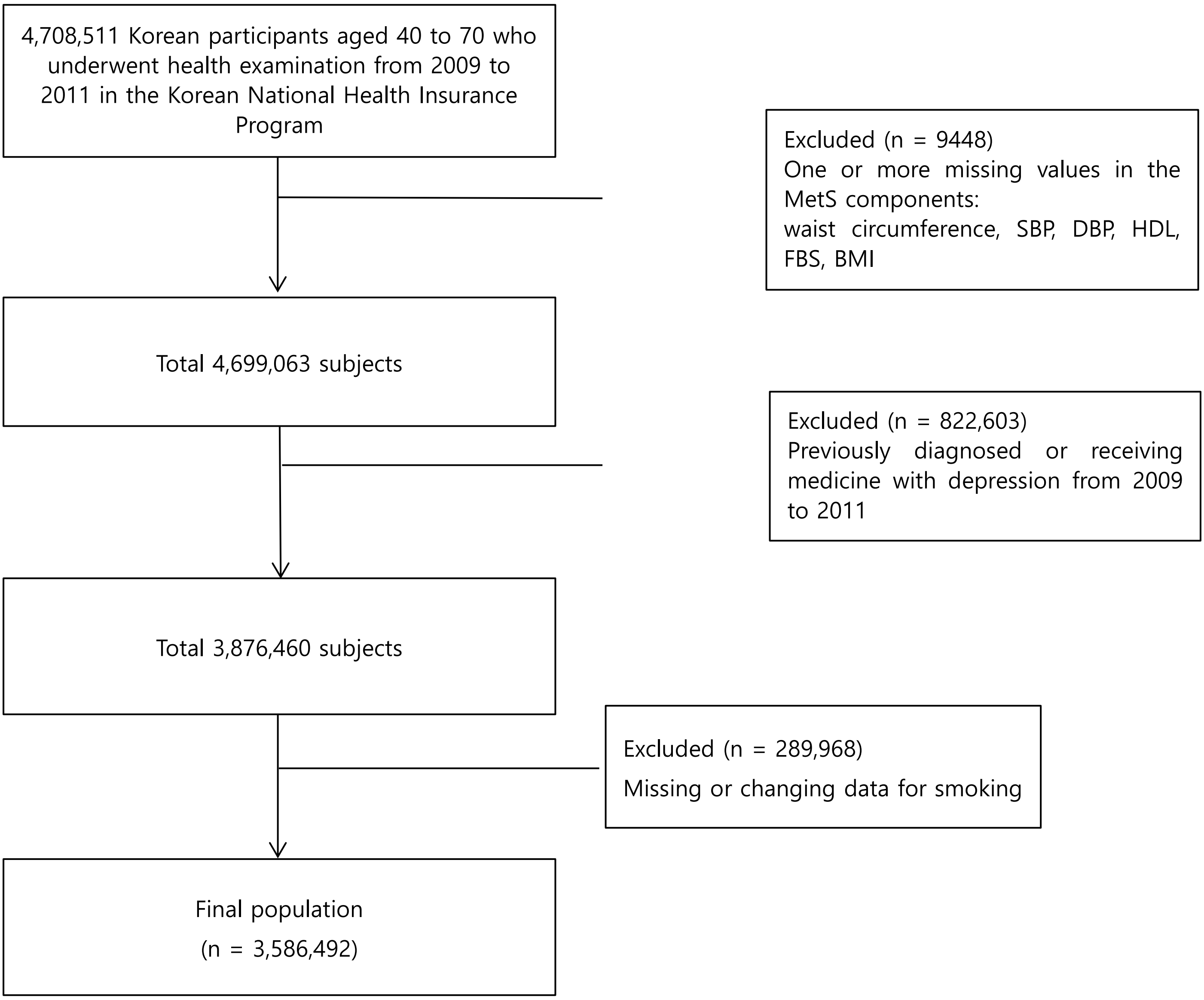

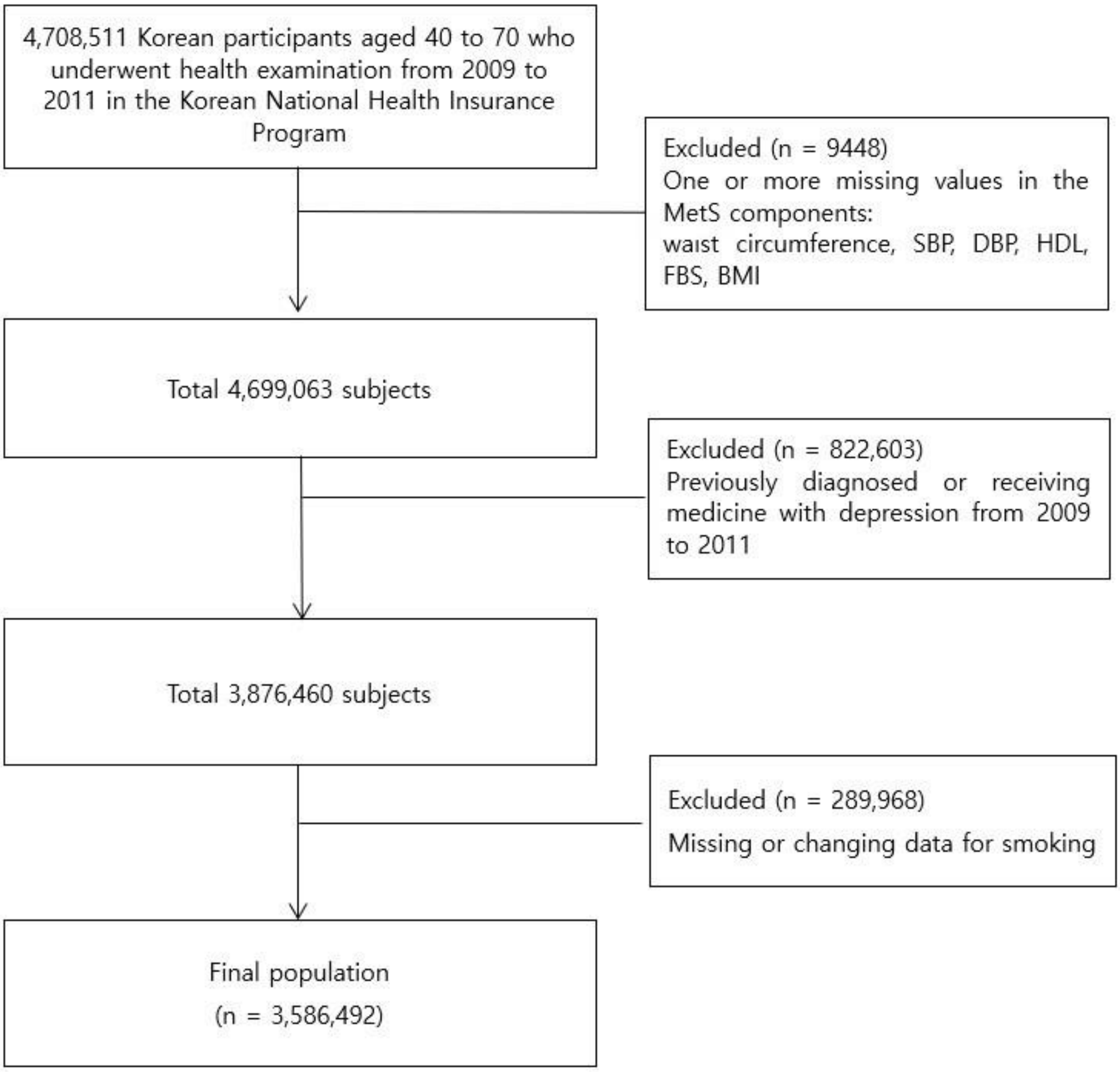
4,708,511 Korean participants aged 40 to 70 who underwent health examination from 2009 to 2011 in the Korean National Health Insurance Program
Excluded (n = 9448)
One or more missing values in the MetS components:
waist circumference, SBP, DBP, HDL, FBS, BMI
Total 4,699,063 subjects
Excluded (n = 822,603)
Previously diagnosed or receiving medicine with depression from 2009 to 2011
Total 3,876,460 subjects
Excluded (n = 289,968)
Missing or changing data for smoking
Final population
(n = 3,586,492)

Supplementary Table S2. Antidepressant code

escitalopram	521101ATD	etizolam	156503ATB
	521102ATD		156502ATB
	474802ATB		156501ATB
	474804ATB		
	474803ATB	bupropion	428102ATR
	474801ATB		428101ATB
fluoxetine	161502ACH	venlafaxine	626402ATR
	161502ATD		626401ATR
			247504ACR
sertraline	227002ATB		247502ACR
	227001ATB		
		milnacipran	355803ACH
paroxetine	209302ATB		355801ACH
	209301ATB		355802ACH
	209304ATR		
	209305ATR	duloxetine	495501ACE
	209304ATE		495502ACE
			495501ATE
fluvoxamine	162502ATB		495502ATE
	162501ATB		
		vortioxetine	628502ATB
imipramine	173701ATB		628503ATB
			628504ATB
amitriptyline	107501ATB		628501ATB
	107504ATB		
	107502ATB	mirtazapine	196201ATB
			196202ATB
trazodone	242901ATB		196204ATB
	242902ATB		196201ATD
	242901ACH		196202ATD
	242903ATR		

Supplementary Table S3. ICD-10 code

F32	Depressive episode
F32.0	Mild depressive episode
F32.1	Moderate depressive episode
F32.2	Severe depressive episode without psychotic symptoms
F32.3	Severe depressive episode with psychotic symptoms
F32.8	Other depressive episodes
F32.9	Depressive episode, unspecified
F33	Recurrent depressive disorder
F33.0	Recurrent depressive disorder, current episode mild
F33.1	Recurrent depressive disorder, current episode moderate
F33.2	Recurrent depressive disorder, current episode severe without psychotic symptoms
F33.3	Recurrent depressive disorder, current episode severe with psychotic symptoms
F33.4	Recurrent depressive disorder, currently in remission
F33.8	Other recurrent depressive disorders
F33.9	Recurrent depressive disorder, unspecified
F34	Persistent mood[affective] disorders
F34.0	Cyclothymia
F34.1	Dysthymia
F34.8	Other persistent mood[affective] disorders
F34.9	Persistent mood[affective] disorder, unspecified
